# Choriocarcinoma Syndrome: A Rare Complication's Impact on Metastatic Germ Cell Tumor Outcomes

**DOI:** 10.7759/cureus.58388

**Published:** 2024-04-16

**Authors:** Sanjeev Herr, Razmig Garabet, Lillian Tseng, Andrea L Harzstark, Michael P Russin

**Affiliations:** 1 Department of Medicine, Drexel University College of Medicine, Philadelphia, USA; 2 Department of Pulmonary Medicine, Kaiser Permanente Northern California, Walnut Creek, USA; 3 Department of Urologic Oncology, Kaiser Permanente Northern California, San Francisco, USA; 4 Department of Oncology, Kaiser Permanente Northern California, Walnut Creek, USA

**Keywords:** case report, tumor lysis syndrome, germ cell tumor, choriocarcinoma, choriocarcinoma syndrome

## Abstract

Choriocarcinoma syndrome is a rare form of tumor lysis syndrome that predominantly occurs in patients with metastatic germ cell tumors, particularly those presenting with extensive lung metastases. We report a case of a previously healthy 37-year-old male who presented with a painless left-sided neck lump and nipples with an increased sensitivity to light touch. Workup revealed a significantly elevated beta-human chorionic gonadotropin, a testicular mass, and innumerable pulmonary metastases, suggesting metastatic non-seminomatous germ cell tumor. Following the initiation of chemotherapy with etoposide, ifosfamide, and cisplatin (VIP), the patient experienced a rapid decline in respiratory function, culminating in acute respiratory distress syndrome and subsequent death from respiratory failure six weeks after starting treatment. This case emphasizes the importance of early detection and intervention in managing non-seminomatous germ cell tumors and highlights the critical need for awareness of choriocarcinoma syndrome's risks, the challenges of treatment delays for fertility preservation, and the exploration of alternative therapeutic strategies to improve outcomes in this high-risk patient population.

## Introduction

Germ cell tumors (GCTs) are relatively rare despite being the most common solid tumor in men between the ages of 15 and 39 years [1]. GCTs are generally highly curable due to chemosensitivity, which has resulted in a five-year survival rate of more than 95% [2]. Choriocarcinoma, an aggressive subtype of GCT, typically presents with widespread lung metastases, intermixed syncytiotrophoblasts and cytotrophoblasts on histopathology, and a high beta-human chorionic gonadotropin (β-hCG) level. Patients who present with choriocarcinoma and extensive lung metastases have demonstrated an increased risk of developing a fatal form of tumor lysis syndrome known as choriocarcinoma syndrome (CS) with subsequent development of diffuse alveolar hemorrhage and acute respiratory distress syndrome (ARDS) [3].

## Case presentation

**Figure 1:**
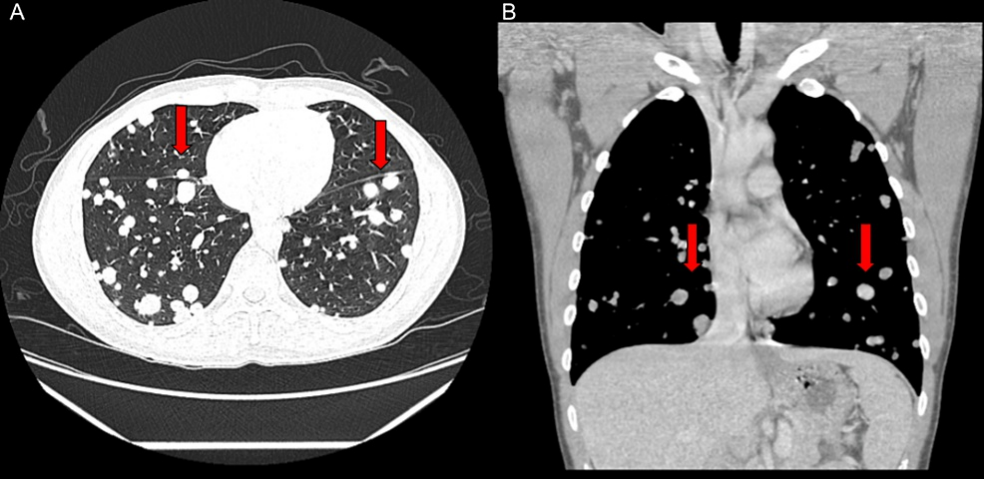
Contrast-enhanced CT scan of the chest in axial (A) and coronal (B) planes demonstrating innumerable pulmonary nodules (red arrows).

A previously healthy 37-year-old male presented to his family physician with a two-day history of painless left-sided neck lump and a one-month history of increased nipple sensitivity to light touch. Initial work-up demonstrated elevated estradiol, β-hCG, prolactin, and lactate dehydrogenase (LDH), and ultrasound of neck mass demonstrated an enlarging lymph node (Table 1). Concern for malignancy prompted the patient to present to the emergency department (ED) the following day for expedited diagnostic investigation. Upon arrival at the ED, the patient also revealed a week-long history of hemoptysis, as well as one day of lower abdominal pain and hematemesis. CT of the neck revealed large, necrotic left supraclavicular level 4 and level 5 lymph nodes, measuring 4.9 cm and 4.6 cm, respectively. CT of the abdomen and pelvis showed enlarged and necrotic precaval and aortocaval lymph nodes, measuring 2.6 cm and 2.3 cm, respectively. CT of the chest demonstrated innumerable pulmonary nodules (Figure 1). The patient was then admitted for further work-up and initiation of chemotherapy.

**Table 1:** Blood investigation abnormalities.

Laboratory test	Patient value	Reference range
Beta-human chorionic gonadotropin	665,570 mIU/mL	<5 mIU/mL
Lactate dehydrogenase	582 U/L	<270 U/L
Estradiol	1,457 pg/mL	<50 pg/mL
Prolactin	582 U/L	2-18 U/L

Physical examination and scrotal ultrasound demonstrated a non-tender right testicular mass that was concerning for primary testicular malignancy. Further staging demonstrated small brain infarcts on brain MRI without evidence of abnormal enhancement to suggest intracranial metastatic disease and also provided a diagnosis of stage IIIC non-seminomatous germ cell tumor (NSGCT). A β-hCG level of 665,570 mIU/mL and an LDH of 676 U/L further categorized it as poor risk. Immediate treatment with four cycles of etoposide, ifosfamide, and cisplatin (VIP) was recommended. Against medical advice, the patient opted to delay treatment for sperm banking and was then discharged with allopurinol for tumor-lysis prophylaxis. A confirmation biopsy was not pursued to expedite care, as the tumor markers and clinical picture were both consistent with NSGCT.

Eight days after diagnosis, the patient returned to the ED with a three-day history of chest pain, dyspnea, and hemoptysis. The patient was then admitted for increased shortness of breath and hypoxemia. On cycle one, day two of VIP treatment, he was transferred to the intensive care unit (ICU) for increased oxygen requirement. The patient then required a high-flow nasal cannula of 40 L/min at a fraction of inspired oxygen (FiO2) of 95% to achieve partial pressure of oxygen (PaO2) of 102 mmHg. CT angiogram of his lungs was negative for pulmonary embolism but demonstrated extensive bilateral airspace opacities, which were new from CT three days prior, before the start of his chemotherapy. The transthoracic echocardiogram did not reveal any cardiac abnormalities. He was treated empirically for pneumonia with broad-spectrum antibiotics, and he received diuretics for noncardiogenic pulmonary edema from ARDS.

Despite these supportive measures, his respiratory disease progressed such that he was intubated one week after the start of his VIP treatment. The patient received only an abbreviated three days of a full five-day course of chemotherapy, given his respiratory failure and rising liver function tests. For his severe hypoxemia, he required intermittent neuromuscular blockade and was ventilated in a prone position for three weeks. He also received rescue inhaled nitrous oxide for two weeks during this time. Five weeks after his abbreviated VIP cycle, the patient entered the late fibrotic phase of ARDS. The development of leukopenia following the initiation of chemotherapy prompted the administration of filgrastim. His lungs' poor compliance resulted in pneumomediastinum from ventilator-associated barotrauma. He was unable to ventilate adequately despite maximum ventilator settings. He ultimately died six weeks after his transfer to the ICU from acute respiratory failure.

## Discussion

Despite GCTs constituting a small percentage of cancers in men, they are the most common solid tumor malignancy in men aged 15-39 years [1]. GCTs are further divided into seminomatous germ cell tumors (SGCT) and nonseminomatous germ cell tumors (NSGCTs). NSGCTs that are considered poor prognosis account for 16% of these malignancies and have shown a five-year survival rate of only 67% [4]. Of those considered with poor prognosis, 10-20% have demonstrated an increased risk of developing choriocarcinoma syndrome, particularly after induction of chemotherapy, which has a high mortality rate [5,6]. Moran-Ribon et al. further described these types of patients as super-high-risk GCTs that clinically presented as a more respiratory distress-type picture with bulky pulmonary disease, very high β-hCG, hypoxemia, and any two of the following symptoms: dyspnea, chest pain, hemoptysis, and weight loss [5]. When it comes to patients with a clinical presentation consistent with poor-risk NSGCT, it is imperative to maintain a high index of suspicion for the development of choriocarcinoma syndrome.

First described by Logothetis in 1984, CS presented in patients with advanced GCTs as hemorrhage from metastatic sites, a high volume of choriocarcinoma elements, and an elevated β-hCG level over 50,000 IU/L [7]. One retrospective study was able to identify potential risk factors for developing CS and found that the greatest indicators were an Eastern Cooperative Oncology Group (ECOG) Performance Status ≥2 and metastatic lung involvement ≥50% [6]. The paucity of literature surrounding the risk factors that lead to this development further emphasizes the importance of being cautious when establishing treatment plans for these patients.

In terms of treatment considerations for these patients, the standard of care for poor-risk NSGCTs involves four cycles of bleomycin, etoposide, and cisplatin combination (BEP). An alternative regimen of ifosfamide, etoposide, and cisplatin (VIP) is used when bleomycin is contraindicated, as was the case with our patient due to their innumerable pulmonary metastases [1]. Massard et al. demonstrated that adopting a reduced induction chemotherapy approach with etoposide and cisplatin (EP) lowered the risk of ARDS; however, it also resulted in a comparatively lower overall rate of long-term survival in contrast to the full regimen induction [8]. Comparatively, Tryakin et al. reported that reduced induction chemotherapy led to a decreased risk of life-threatening complications without a significant change in long-term survival [9]. The juxtaposition of this outcome data underscores the need for further investigation into alternative treatment regimens for these patient populations.

Some important things to further consider are the time to initiate care and the impact of delaying treatment on the outcome of these patients as well as the various causes of delayed care that may be encountered. Given the age of these patients, one must consider the impact of a desire to participate in fertility preservation prior to initiation of chemotherapy and the need to expedite sperm banking. In this case and a case reported by Zeitjian et al., patients delayed induction of treatment for sperm banking and presented with worsening symptoms in the form of massive hemoptysis eight and 16 days later, respectively [10]. Even when expedited sperm banking is available, as it was in this case, the difficulty of banking with the pressure of a new diagnosis and significant symptoms can lead to delays. Furthermore, each aspect of delay to care creates the potential for a cascading effect. A slight delay in sperm banking can lead to a further delay due to staffing capabilities for chemotherapy initiation and so forth. Future research should be aimed not only at the optimal therapy for patients at risk for developing CS but also at ways to mitigate delays.

## Conclusions

Choriocarcinoma syndrome remains a formidable challenge in the treatment of metastatic GCTs and this case contributes to the scant literature on choriocarcinoma syndrome. The details of this case further demonstrate the necessity of early detection and prompt tailored therapeutic interventions to improve survival outcomes. This places an emphasis on the importance of developing a further understanding of contributing factors to treatment delay in patients. Overall, our report seeks to underscore the necessity for ongoing research into optimal management strategies for patients with high-risk GCTs.
